# Unpacking the Relationships between Impulsivity, Neighborhood Disadvantage, and Adolescent Violence: An Application of a Neighborhood-Based Group Decomposition

**DOI:** 10.1007/s10964-017-0695-3

**Published:** 2017-05-29

**Authors:** Matt Vogel, Maarten Van Ham

**Affiliations:** 1 Department of Criminology and Criminal Justice, University of Missouri—St. Louis, St. Louis, USA; 2 Faculty of Architecture and the Built Environment, OTB—Research for the Built Environment, Delft University of Technology, Delft, The Netherlands; 3 School of Geography and Geosciences, University of St. Andrews, St Andrews, UK

**Keywords:** Person-context research, Neighborhood effects, Decomposition, Delinquency

## Abstract

Scholars have become increasingly interested in how social environments condition the relationships between individual risk-factors and adolescent behavior. An appreciable portion of this literature is concerned with the relationship between impulsivity and delinquency across neighborhood settings. The present article builds upon this growing body of research by considering the more nuanced pathways through which neighborhood disadvantage shapes the development of impulsivity and provides a situational context for impulsive tendencies to manifest in violent and aggressive behaviors. Using a sample of 12,935 adolescent from the National Longitudinal Study of Adolescent to Adult Health (Add Health) (mean age = 15.3, 51% female; 20% Black, 17% Hispanic), we demonstrate the extent to which variation in the association between impulsivity and delinquency across neighborhoods can be attributed to (1) differences in mean-levels of impulsivity and violence and (2) differences in coefficients across neighborhoods. The results of a series of multivariate regression models indicate that impulsivity is positively associated with self-reported violence, and that this relationship is strongest among youth living in disadvantaged neighborhoods. The moderating effect of neighborhood disadvantage can be attributed primarily to the stronger effect of impulsivity on violence in these areas, while differences in average levels of violence and impulsivity account for a smaller, yet nontrivial portion of the observed relationship. These results indicate that the differential effect of impulsivity on violence can be attributed to both developmental processes that lead to the greater concentration of violent and impulsive adolescents in economically deprived neighborhoods as well as the greater likelihood of impulsive adolescents engaging in violence when they reside in economically disadvantaged communities.

## Introduction

Over the past two decades, research has increasingly highlighted the importance of social context for adolescent development and well-being. Much of this research has focused on the ways in which school and neighborhood environments influence outcomes like school performance (Dotterer and Lowe 2011; Irvin et al. 2011), mental health (Nair et al. 2013), and delinquent behavior (Deutsch et al. 2012; Vogel et al. 2015). On the whole, findings from this body of literature indicate that contextual risk-factors are robust and persistent correlates of youth behavior. More recently, scholarly attention has shifted to understanding the ways in which social environments condition the relationships between individual risk-factors and adolescent behavior, especially delinquent and violent conduct. This emerging perspective, referred to here as “person-context research”, assumes that behavioral outcomes are not the result of individual or environmental factors, but are dependent on who is in what environment (Messner and Zimmerman 2012; Wikström 2004). The general consensus is that dispositional risk factors, such as impulsivity or low self-control, are contingent on the characteristics of broader ecological contexts, such as the school one attends or neighborhood in which one resides (e.g., Fine et al. 2016; Lynam et al. 2000; Vogel and Barton 2013; Zimmerman 2010).

Much of the research in this area has focused on the differential effects of impulsivity across neighborhood settings (see Vaughan 2017 for recent overview). Although there remains some debate as to the exact parameters of the association, much of the empirical literature demonstrates that structural characteristics and the social processes at work in disadvantaged neighborhoods moderate the influence of impulsivity on criminal behavior (c.f. Vazsonyi et al. 2006; Zimmerman et al. 2015). These differential effects, sometimes referred to as evidence of “contextual moderation”, are often attributed to neighborhood features providing greater access to criminogenic opportunities or greater exposure to socialization processes promoting violence over normative behaviors.

Two observations complicate the results presented in prior research. First, a sizable body of literature suggests that indicators of neighborhood deprivation are associated with youth offending; adolescents who reside in economically deprived neighborhoods typically exhibit higher levels of problem behaviors than adolescents from more affluent neighborhoods (see Kubrin and Weitzer 2003; Sampson 2002 for reviews). Second, emerging research suggests that the social processes in disadvantaged neighborhoods may contribute to the development of undesirable personality traits (Hart et al. 2008; Pratt et al. 2004; Turner et al. 2005). From this vantage point, economic deprivation, limited informal control, and socialization processes promoting crime and delinquency may place youth from disadvantaged neighborhoods at a higher risk of developing, for instance, impulsive tendencies. As a result, these youth typically exhibit higher levels of delinquency and higher levels of impulsivity than those from more affluent communities. It remains unclear whether evidence of contextual moderation uncovered in prior research reflects a “true” neighborhood effect or developmental processes that give rise to compositional differences in both impulsivity and delinquency across neighborhoods. In other words, whether impulsive youth are more likely to offend when they live in impoverished areas, or whether youth who live in impoverished areas are more likely to display impulsive and aggressive tendencies. As is argued below, disentangling contextual influences from developmental processes is critical to understanding the complex role that neighborhoods play in adolescent behavior.

The present article attempts to bridge this gap in the literature in several key regards. The analyses begin by examining the relationship between impulsivity and violent behavior among a nationally representative sample of American adolescents. Census data are linked to the residential tracts of survey respondents to examine whether and how indicators of neighborhood disadvantage moderate the relationship between impulsivity and self-reported violence. Finally, a neighborhood-based, group decomposition framework is used to partition the moderating effect of neighborhood context into its constituent parts. This technique assumes that developmental and contextual factors lead to unique data generating processes that differentially affect the estimation of contextual moderation. While the discussion is framed around recent research on impulsivity and neighborhood disadvantage, these techniques can be applied to a broad range of topics linking individuals to broader ecological contexts. And, although the procedures presented here have been used in most social science disciplines, with a few exceptions, they have yet to be embraced in person-context research.

Person-context research assumes that behavioral risk factors are more strongly associated with criminal behavior in particular social contexts (for a comprehensive overview see Messner and Zimmerman 2012). An appreciable portion of this research has focused on identifying neighborhood-level mechanisms that either mitigate or exacerbate the association between impulsivity and delinquency (see Vaughan 2017 for a comprehensive review). The overwhelming focus on impulsivity, or low self-control, is likely attributed to the fact it is one of the most robust and well-studied dispositional correlates of delinquency (Gottfredson and Hirschi 1990; Pratt and Cullen 2000). Several studies have reported the effect of impulsivity on offending to be strongest among adolescents living in economically disadvantaged neighborhoods and neighborhoods characterized by low levels of adult supervision (Jones and Lynam 2009; Lynam et al. 2000; Meier et al. 2008; Vogel 2016). Other studies have uncovered the opposite—that the effect of impulsivity is stronger in relatively affluent neighborhoods with high levels of collective efficacy (Fine et al. 2016; Wikström and Loeber 2000; Zimmerman 2010). And, at least two studies have failed to detect any evidence of contextual moderation (Vazsonyi et al. 2006; Zimmerman et al. 2015).

While research in this vein has produced somewhat equivocal findings, each of these studies is grounded within a similar theoretical framework and each points to similar mechanisms purported to underlie the stronger effect of impulsivity on delinquency in certain neighborhoods—namely, the differential distribution of contextual risk-factors. For instance, Lynam and colleagues (2000) draw from routine activities theory (Cohen and Felson 1979; Osgood et al. 1996; Osgood and Anderson 2004) and posit that the lack of informal social control in disadvantaged neighborhoods provides greater opportunity for impulsive individuals to engage in rule violating behavior. Zimmerman (2010), on the other hand, argues that disadvantaged neighborhoods present a variety of risk-factors for delinquency that suppress the influence of dispositional risk factors. When these external factors are removed, the association between impulsivity and delinquency emerges more clearly. In this case, contextual risk-factors in the most disadvantaged areas may push all youth to engage in criminal conduct. In relatively low-risk contexts, youth with the strongest internal controls may benefit the most from the resources available to them (see also, Fine et al. 2016; Vaughan 2017). Finally, Vazsonyi and colleagues (2006) draw from Gottfredson and Hirschi (1990) and argue that opportunities for crime are ubiquitous, thus explaining the lack of moderation uncovered in their analysis.

**Fig. 1:**
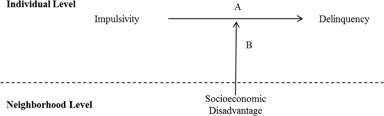
Hypothesized moderation association neighborhood disadvantage on impulsivity-delinquency. **a** Direct effects of impulsivity on delinquency. **b** Moderation effect of disadvantage on the impulsivity-delinquency association

Figure 1 presents a theoretical diagram outlining the hypothesized moderating relationship of neighborhood disadvantage on the association between impulsivity and delinquency typically explored in person-context research. Path A represents the direct effect of impulsivity on offending, and Path B represents the moderating role of neighborhood disadvantage. The dotted line differentiates processes hypothesized to occur at the individual level from those at the neighborhood level—in this case, path A reflects an individual-level relationship, while path B reflects the moderating role of neighborhood-level processes.

Drawing from the neighborhood effects literature, an alternative explanation for a stronger effect of impulsivity, or dispositional risk factors more generally, in economically deprived neighborhoods can be attributed to the fact that high-risk individuals are often overrepresented in high-risk environments. In traditional thinking on selection effects, this means that people with particular background characteristics differentially select certain types of neighborhoods; for instance, poor people may be more likely to live in economically deprived neighborhoods as housing prices are lower (van Ham and Manley 2012; van Ham et al. 2012). However, much of the person-context literature focuses on adolescents and it bears to reason that the non-random distribution of children and adolescents across neighborhoods overwhelmingly reflects the decisions of parents. While it is unlikely that impulsive youth choose to live in neighborhoods with high levels of socioeconomic disadvantage, as it is their parents who make residential decisions, it is not unreasonable to assume that family and broader community characteristics associated with neighborhood disadvantage contribute to the greater likelihood that these children develop impulsive traits.

Building from Wikstrom and Sampson (2003), community context may contribute to adolescent behavior through two complimentary processes: (1) neighborhoods can affect the presence of situational opportunities in which crime is considered a reasonable option and (2) neighborhoods, through both direct and indirect means, can influence the development of criminal predispositions, such as low self-control or impulsivity. In regards to the former (presence of situational opportunities), neighborhood disadvantage may diminish informal social control and provide greater opportunity for adolescents to engage in unstructured activities with their peers, away from adult chaperones—prime conditions for delinquency (Bernasco et al. 2013; Hoeben and Weerman 2014; Weerman et al. 2015; Wikström and Butterworth 2006). In this sense, neighborhoods can be seen as having a direct influence on individual behavior—sometimes referred to as a “neighborhood” or “contextual” effect.

In regards to the latter (development of criminal dispositions), neighborhoods can be thought of as a collective form of socialization, whereby the shared monitoring and supervision of youth behavior within the larger community framework helps shape healthy child development (Leventhal and Brooks-Gunn 2000; Pratt et al. 2004; Sampson 2002; Shaw and McKay 1942). Disadvantaged neighborhoods, characterized by low levels of cohesion and limited communication among neighbors, may be less adept at creating self-control in children. Moreover, families living in economically deprived communities may face a number of disadvantages, such as single-earner families, unemployment, and poverty, which detract from their ability to adequately socialize their children. Coupled with the absence of community resources to alleviate the burden, children growing up in these areas may experience inconsistent supervision, inconsistent rule enforcement, and inconsistent discipline when they misbehave. As a result of both community and family socialization practices, children may not develop the same executive functions (e.g., the ability to delay gratification) as children from more affluent communities (Hart et al. 2008). Indeed, several studies have reported an inverse relationship between neighborhood disadvantage and levels of self-control, in some cases rivaling the effects of family socialization (Pratt et al. 2004; Turner et al. 2005; but see Gibson et al. 2010). In this sense, neighborhood disadvantage may contribute to the development of criminogenic traits like impulsivity. It bears to reason that these developmental processes will be stratified by place, leading to a greater concentration of impulsive adolescents in certain areas than others. These differences are likely to be differentially distributed across levels of socioeconomic disadvantage, such that the most high-risk youth are disproportionately clustered into the most high-risk environments.

**Fig. 2:**
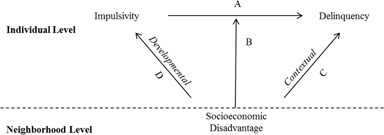
Hypothesized direct and moderation effects of neighborhood disadvantage on impulsivity, delinquency, and the relationship between impulsivity and delinquency. **a** Direct effects of impulsivity on delinquency. **b** Moderation effect of disadvantage on the impulsivity-delinquency association. **c** Direct effect of neighborhood disadvantage on delinquency. **d** Direct effect neighborhood disadvantage on impulsivity

Figure 2 presents an expanded theoretical model of the moderating role of neighborhood disadvantage on the association between impulsivity and delinquency, incorporating the role of developmental and contextual influences. In this figure, Path C represents the direct effect of neighborhood-level disadvantage on offending. This pathway is assumed in most person-context research and can be directly assessed through the main effect of neighborhood disadvantage in standard regression models. Path D reflects the developmental processes that may lead to higher levels of impulsivity among adolescents who grow up in disadvantaged communities. Unlike the direct effect of neighborhood disadvantage, this pathway is rarely considered and its influence cannot be gleaned from a standard regression model. Thus, to truly understand the moderating role of neighborhood context on the association between impulsivity and offending, researchers need not only examine paths A and B, but also need to carefully consider the role of C and D.

## Current Study

The purpose of the current study is to examine the more nuanced model of impulsivity, neighborhood disadvantage, and self-reported violence presented in Fig. 2 among a nationally representative sample of American adolescents. Drawing from the theoretical processes highlighted in Fig. 2, the analyses begin by examining the independent associations between impulsivity and neighborhood disadvantage on self-reported violence. We hypothesize that impulsivity (Hypothesis 1) and neighborhood disadvantage (Hypothesis 2) will be positively associated with self-reported violence. The analyses next assess whether the relationship between impulsivity and self-reported violence is contingent on levels of neighborhood disadvantage. Consistent with the work of Vogel (2016) using the same data, we anticipate that neighborhood disadvantage will strengthen the relationship between impulsivity and violence, implying a positive interaction effect (Hypothesis 3). The final set of analyses examine whether differences in average levels of violence and impulsivity across communities can help explain variation in the effect of impulsivity on violence across neighborhoods with varying levels of socioeconomic disadvantage. While it is more difficult to anticipate the exact nature of the mechanisms driving the hypothesized interaction, the theoretical processes presented in Fig. 2 portend that average levels of both violence and impulsivity will be higher in disadvantaged communities. These compositional differences should then partially explain the moderating effect of neighborhood disadvantage on the association between neighborhood disadvantage and self-reported violence (Hypothesis 4).

## Methods

### Data

Data for the analyses were drawn from the National Longitudinal Study of Adolescent to Adult Health (Add Health), a nationally representative survey of adolescents enrolled in high school during the 1994–95 academic year and followed through early adulthood (with data collection ongoing). The original survey design included a sample of 80 high schools and 52 middle schools from the United States with an unequal probability of selection, ensuring representativeness with respect to region of country, urbanicity, school size, school type, and ethnicity. In the first phase of data collection, a brief questionnaire was administered to all youth enrolled in grades 7–12 in each of the 132 schools with no make-up given for absent students. The in-school survey covered topics such as socio-demographic characteristics, risk behaviors, future expectations, health status, perceived school climate, and household structure. In addition to these data, school administrators provided information on characteristics such as graduation rate, retention rate, and class size.

From the initial in-school survey, over 20,000 students were selected to participate in the first wave of the longitudinal follow-up study. The Wave I data included 39 self-report questionnaires on topics covering general health, romantic relationships and contraception, employment and income, as well as personality characteristics and delinquent behavior. Additionally, respondents’ home addresses were geocoded, and geographic information from the 1990 census is available at the block group, tract, and county level for each respondent. During the following year (1995–96), respondents who were still in high school were asked to complete a second wave of questionnaires. These data included information from roughly 14,000 respondents (excluding those who were high school seniors in Wave I). The present analysis draws on a sample of 12,935 respondents who participated in the first two waves of the survey, spanning the years 1994–1996.

### Violence

*Violence*, the primary dependent variable, is a count-based measure of the number of the following acts the respondent in which the respondent engaged during the 12 months prior to the Wave 2 interview: (1) injuring someone badly enough to need medical attention, (2) shooting or stabbing someone, (3) using or threatening to use a weapon to get something from someone (4) participating in a group fight, (5) using a weapon in a fight, (6) pulling a knife or gun on someone, (7) getting into a serious physical fight. The scale was constructed by first dichotomizing each of these seven items, then summing across items to generate count-based measure that captures the variety of violent offenses endorsed by Add Health respondents (alpha = 0.93).

### Impulsivity

*Impulsivity* is measured by the extent to which respondents agreed with the following statement: when making decisions, you usually go with your “gut feeling” without thinking too much about the consequences of each alternative. Responses to this item are arranged along a five item Likert scale ranging from strongly disagree (low impulsivity) to strongly agree (high impulsivity). This item closely resembles (a lack of) premeditation, one of the four key facets of impulsivity proposed by Whiteside and Lynam (2001).^1^

### Neighborhood Socioeconomic Disadvantage

*Neighborhood disadvantage* is measured as a standardized index of the percent of a respondents neighborhood receiving welfare, the percent living at or below poverty, the percent unemployed, and percent of female headed households (alpha = 0.923). It is coded such that higher values reflect a greater degree of socioeconomic disadvantage.

### Race

Race differentiates respondents who identified as non-Hispanic white (55%), non-Hispanic black (20%), Hispanic (17%), and non-Hispanic other race (8%).

### Age

Age is measured in years at the time of the Wave 1 interview (Mean = 15.1).

### Sex

Sex is a dichotomous variable differentiating males from female (female = 1; 51%).

### Two Parent Household

Two parent household is a dichotomous variable differentiating respondents living with both biological parents from any other family configuration (both parents = 1; 55%).

### Analytic Strategy

The conventional approach to assessing neighborhood moderation in person-context research involves estimating a regression model in which neighborhood characteristics, impulsivity, and their product term are included alongside a series of control variables to predict some form of delinquency. The regression equation takes on the basic form:^2^

1 $$Y = a + {b_1}{X_1} + {b_2}{X_2} + {b_3}{X_1}{X_2}$$

Where Y refers to a scale of delinquency, b_1_ refers to the slope of impulsivity (denoted by Path A in Fig. 2), b_2_ refers is the slope of neighborhood disadvantage (Path C), and b_3_ is the product term for the neighborhood disadvantage * impulsivity (Path B). A significant coefficient for b_3_ is usually considered sufficient evidence of contextual moderation, and is often interpreted as the expected change in the slope of b_2_ across levels of neighborhood disadvantage—in other words, how neighborhood context mitigates or exacerbates the effect of impulsivity on offending.

This interpretation is problematic, as the interaction term, in part, reflects compositional differences in impulsivity across neighborhoods (due to relationship D in Fig. 2). For the reasons outlined above, we might expect average levels of both impulsivity and violence to be higher among youth residing in economically disadvantaged neighborhoods. This suggests that Y and X_1_ will increase with neighborhood disadvantage. In the traditional regression framework, these higher averages can generate a statistically significant coefficient for b_3_ without any true difference in the slope of b_1_ across neighborhoods. Thus, to sufficiently make claims about contextual moderation, we need to rule out the possibility that the observed coefficient is not driven by higher averages levels of impulsivity and delinquency alone (as demarcated in paths C and D in Fig. 2).

The issue of developmental vs. contextual effects can be viewed as a special version of a more general problem identified in the social science literature: identifying the extent to which differences in rates across groups reflects differences in group composition. While these techniques have been utilized in other disciplines, they have yet to be employed in person-context research. Oaxaca (1973) and Blinder (1973) independently proposed a relatively straightforward means to address this problem, as it applied to gender differences in earnings. The same basic approach also applies here. In the standard framework, group-based differences can be attributed to two factors—differences in levels and differences in slopes. In the earnings nomenclature, this means that a difference in income between males and females could reflect, in part, higher average education among males (levels) and the portion that cannot be explained by educational differences (e.g., the unexplained portion) would then be attributed to a true interaction effect.^3^ In the case of neighborhood context and impulsivity, the differences in levels can be viewed as analogous to the differential distribution of individual risk-factors across neighborhoods (Paths C and D), while the “unexplained” part of the interaction could be interpreted as “neighborhood moderation” (Path B).

In its simplest application, the decomposition involves a four-step process. In the first step, we followed prior research in this area (e.g. Farrington and Loeber 2000; Fine et al. 2016; Graif 2015; Vogel 2016) and collapsed the neighborhood disadvantage index at the 75th percentile to create a dichotomy differentiating “disadvantaged” neighborhoods from all other neighborhoods. Second, differences in average levels of impulsivity and offending were assessed by comparing means across neighborhood groupings. Third, two separate regression equations were estimated, one for respondents living in disadvantaged neighborhoods, and the second for respondents living in all other neighborhoods such that:

2 $${\hat y_H} = {a_H} + {b_H}{X_H}$$

and

3 $${\hat y_L} = {a_L} + {b_L}{X_L}$$

In these equations *ŷ* is the predicted level of self-reported violence, *a* is the regression constant, *X* is the mean level of impulsivity, and *b* is the regression coefficient. The subscript *H* refers to respondents living in neighborhoods with high levels of disadvantage and *L* refers to respondents living in neighborhoods with low levels of disadvantage. Similar to Eq. 1, contextual moderation can be assessed by comparing *b*_*H*_ and *b*_*L*_, in this case, the Clogg Test for the equality of coefficients can be used to assess statistical significance (Paternoster et al. 1998).^4^ The difference in average levels of self-reported delinquency can then be expressed as the difference in predicted levels of delinquency between Eqs. 2 and 3:

4 $$\left( {{{\hat y}_H} - {{\hat y}_L}} \right) = \left( {{a_H} + {b_H}{{\bar x}_H}} \right) - \left( {{a_L} + {b_L}{{\bar x}_L}} \right)$$

Which can be expanded into the Blinder (1973) and Oaxaca (1973) decomposition such that:

5 $$\left( {{{\hat y}_H} - {{\hat y}_L}} \right) = {b_H}\left( {{{\bar x}_H} - {{\bar x}_L}} \right) + {\bar x_L}\left( {{b_H} - {b_L}} \right)$$

In this equation (ŷ_H_–ŷ_L_) is the expected difference in self-reported violence between adolescents living in disadvantaged and non-disadvantaged neighborhoods. b_H_(x̅_H_–x̅_L_) represents the portion of the difference in violence across neighborhoods that can be attributed to compositional differences—in this case, higher average levels of impulsivity in disadvantaged neighborhoods. The final component, x̅_L_(b_H_–b_L_), is the “unexplained” part of the interaction effect, in this case, the portion of the interaction that can be attributed to a stronger effect of impulsivity on violence in disadvantaged neighborhoods.

The two component model can be expanded slightly such that:

6 $$\begin{array}{ccccc}\left( {{{\hat y}_H} - {{\hat y}_L}} \right) = &{b_L}\left( {{{\bar x}_H} - {{\bar x}_L}} \right) + {\bar x_L}\left( {{b_H} - {b_L}} \right) \hfill \\ &+ \left( {{b_H} - {b_L}} \right)\left( {{{\bar x}_H} - {{\bar x}_L}} \right)\hfill \\ \end{array}$$

In this case, the difference in violence across neighborhoods is decomposed into three components, the difference in mean levels of impulsivity [b_L_(x̅_H_–x̅_L_)], the difference in coefficients [x̅_L_(b_H_–b_L_)], and a third component that accounts for the part of the difference that can be attributed to the interaction between levels and coefficients [(b_H_–b_L_) (x̅_H_–x̅_L_)] (Daymont and Andrisani 1984). This third component, as discussed in greater detail below, overcomes scaling issues in X.

Finally, the equations presented in Eqs. 5 and 6 can be expanded slightly to determine the extent to which to which differences in Y (violence), in addition to differences in x̅ (impulsivity), affect the observed interaction. This yields a four component solution initially proposed by Jones and Kelley (1984):

7 $$\begin{array}{ccccc}\\  \left( {{{\hat y}_H} - {{\hat y}_L}} \right) = & \left( {{a_H} - {a_L}} \right) + {b_L}\left( {{{\bar x}_H} - {{\bar x}_L}} \right) + {\bar x_L}\left( {{b_H} - {b_L}} \right) \hfill \\ & + \left( {{b_H} - {b_L}} \right)\left( {{{\bar x}_H} - {{\bar x}_L}} \right)\hfill \\ \end{array}$$

Here [(a_H_–a_L_)] is the difference in the adjusted intercepts of the two groups or the proportion of the observed interaction that can be attributed to variation in mean levels of violence (Y) across neighborhoods. [b_L_(x̅_H_–x̅_L_)] is the component attributed to differences in levels of impulsivity, or how the impact of impulsivity on violence for someone living in a high disadvantage neighborhood would change if they were living in a low disadvantage neighborhood. x̅_L_(b_H_–b_L_) is the difference in the effect on impulsivity on violence across neighborhoods and [(b_H_–b_L_) (x̅_H_–x̅_L_)] is the residual component interpreted as the difference in the interaction between mean levels of impulsivity and coefficients across neighborhoods.^5^^,^ 6 Examining each of these components as a proportion of the raw difference in predicted levels of violence, (ŷ_H_−ŷ_L_), provides a means to quantify the contribution of compositional differences in violence and impulsivity to the observed moderating effect of neighborhood disadvantage on the association between the two. Thus, the third component provides the estimate of neighborhood moderation (Path B in Fig. 2) and the first and second components provide the estimates of developmental processes that give rise to compositional differences across neighborhoods (paths C and D).

## Results

### Main Results

Table 1 presents the descriptive statistics for the variables included in the analysis. The analytic sample was approximately 51% female, 20% non-Hispanic black, 17% Hispanic, and 8% non-Hispanic other race. The average age was 15.3 years, and 55% of respondents reported living with both biological parents. Respondents reported an average level of impulsivity of 3.02 on a five-point scale.

**Table 1:** Descriptive statistics (*N* = 12,935)

	Mean (Prop)	SD	Min	Max
Age	15.31	1.6	12	19
Female	0.51	–	0	1
NH Black	0.20	–	0	1
Hispanic	0.17	–	0	1
NH Other	0.08	–	0	1
Two parent household	0.55	–	0	1
Impulsivity	3.02	1.12	1	5
Neighborhood disadvantage	0.00	0.87	−1.12	3.87

Table 2 presents the results from a regression model in which self-reported violence was regressed on the control variables, impulsivity, neighborhood disadvantage, and the interaction of impulsivity*neighborhood disadvantage. Consistent with some prior research (e.g., Jones and Lynam 2009; Lynam et al. 2000; Meier et al. 2008; Vogel 2016), impulsivity was associated with higher levels of offending, and neighborhood disadvantage moderated this association, such that effect of impulsivity on offending was amplified at higher levels of neighborhood disadvantage. This provides confirmation Hypotheses 1 and 3.

**Table 2:** Regression of self-reported violence on impulsivity, neighborhood disadvantage, and interaction (*N* = 12,935)

	Model 1		Model 2	
	β	se	β	se
Intercept	1.34	0.17***	1.14	0.17***
Age	−0.07	0.01***	−0.06	0.01***
Female	−0.28	0.02***	−0.28	0.02
NH Black	0.12	0.06*	0.12	0.06*
Hispanic	0.18	0.06**	0.18	0.06**
NH Other	0.03	0.07	0.03	0.07
Intact family	−0.10	0.03***	−0.10	0.03***
Neighborhood disadvantage	0.03	0.02	0.03	0.02
Impulsivity	0.06	0.01***	0.06	0.01***
Dis*Impulsivity	–	–	0.03	0.01**

*p < 0.05; **p < 0.01; ***p < 0.001

Table 3 presents the results of the regression models estimated separately for respondents living in neighborhoods at the top-quartile of socioeconomic disadvantage and respondents living in all other neighborhoods. Consistent with the models presented in Table 2, the effect of impulsivity on self-reported violence is stronger among respondents living in disadvantaged neighborhoods relative to those living in more affluent neighborhoods (relationship B in Fig. 2; [Z = 1.92; one-tailed test]). Predicted levels of self-reported violence in disadvantaged and non-disadvantaged neighborhoods were next generated by substituting the mean level of impulsivity into Eqs. 2 and 3, respectively. This yields the constituent terms for Eq. 7. For instance, the mean level of impulsivity in disadvantaged neighborhoods was 3.09; substituting this value into the regression equation (holding the other covariates constant) provides a ŷ of 1.689 [ŷ = a + bx̅]. Table 4 presents the summary statistics from these subsequent regressions.

**Table 3:** Regression of self-reported violence on impulsivity across neighborhood type (*N* = 12,935)

	Disadvantaged tracts		Non-disadvantaged tracts	
	β	se	β	se
Intercept	1.34	0.33***	1.31	0.18***
Age	−0.06	0.02***	−0.72	0.03***
Female	−0.26	0.07***	−0.29	0.03***
NH Black	0.09	0.07	0.20	0.08*
Hispanic	0.28	0.10*	0.16	0.05**
NH Other	0.26	0.19	0.06	0.03
Intact family	−0.21	0.06***	−0.07	0.03*
Impulsivity	0.11	0.03***	0.04	0.02**

*p < 0.05; **p < 0.01; ***p < 0.001

**Table 4:** Summary statistics of impulsivity and violence across neighborhood type

	Disadvantaged tracts	Non-disadvantaged tracts	Difference
Mean impulsivity	3.093	2.998	0.095
Impulsivity slope	0.112	0.043	0.069
a	1.342	1.305	0.037
Y-hat	1.689	1.434	0.255

The results of the decomposition analysis indicate that roughly 81% of the observed interaction between impulsivity and neighborhood disadvantage can be attributed to differences in the slope of impulsivity across neighborhoods (Table 5). Compositional differences in self-reported violence (14%—Path C in Fig. 2) and impulsivity (2%—Path D in Fig. 2) make up the remainder of the observed interaction. Collectively, these findings suggest that much of the observed interaction can be attributed to contextual moderation—that is, that the effect of impulsivity on violence is stronger in socioeconomically disadvantaged neighborhoods. However, a non-trivial proportion, just under 16%, can be attributed to the differential concentration of high-risk (i.e., violent and impulsive) adolescents in disadvantaged neighborhoods.

**Table 5:** Decomposition of difference in impulsivity—violence association across neighborhood type

Component		Raw	Percent
Total difference	(ŷ_H_–ŷ_L_)	0.255	100.0%
Portion explained by differences in mean violence	(a_H_–a_L_)	0.037	14.5%
Portion explained by differences in mean impulsivity	b_L_(x̅_H_–x̅_L_)	0.004	1.6%
Portion explained by differences in impulsivity slope	x̅_L_(b_H_–b_L_)	0.207	81.2%
Residual difference	(b_H_–b_L_)(x̅_H_–x̅_L_)	0.007	2.7%

### Sensitivity Analyses

While consistent with much prior research (e.g., Farrington and Loeber 2000; Fine et al. 2016; Graif 2015; Vogel 2016) and necessary for the decomposition procedure, the decision to dichotomize the measure of neighborhood disadvantage at the 75th percentile may be viewed as somewhat arbitrary. As sensitivity analyses, the regression models and corresponding decomposition analyses were re-estimated by shifting the designation of ‘disadvantaged neighborhoods’ to the 90 percentile. The results of these supplemental analyses can be found in Tables 6 and 7. Although the parameter estimates and corresponding components vary from those presented in the preceding tables, the general conclusions remain the same. The majority of the interaction between impulsivity and violence can be attributed to a true difference in slopes; however, an appreciable portion of the interaction can be attributed to higher levels of impulsivity and violence among adolescents residing in the most disadvantaged areas. Thus, the cut-point at which neighborhoods were deemed “disadvantaged” had little substantive bearing on the results presented here.

## Discussion

Over the past several decades, scholars have become increasingly attuned to the importance of social context in the etiology of adolescent development and behavior. This research has highlighted how chacteristics of schools and neighborhoods contribute to, for instance, educational achievement (Irvin et al. 2011; Dotterer and Lowe 2011), relationship conflict (Foshee et al. 2013), mental health (Nair et al. 2013), and prosocial adjustment (Riina et al. 2013). A parallel body of literature has emphasized how social and spatial environments condition the relationships between individual risk-factors and adolescent behavior (e.g., Deutsch et al. 2012; Zimmerman 2010). This research demonstrates that adolescent behavior cannot be attributed to dispositional or contextual factors alone. Instead, behaviors such as drug use, violence, and delinquency are best understood through the confluence of individual and environmental risk-factors. While recent research in this vein continues to underscore the intricate linkages between individual and contextual risk-factors and their attendant consequences for adolescent behavior (e.g., Chen and Vazsonyi 2013; Jensen et al. 2017; Vogel et al. 2015; Zimmerman and Farrell 2017), comparatively fewer studies have focused on the more complex pathways through which these processes operate. The present study attempted to bridge this gap in the literature by examining the relationship between impulsivity (a dispositional risk-factor) and violence among respondents living in neighborhoods characterized by varying degree of socioeconomic disadvantage (a contextual risk-factor). While some prior research in this area indicates the association between impulsivity and offending to be strongest in economically deprived communities (e.g., Lynam et al. 2000; Vogel 2016; c.f., Fine et al. 2016; Zimmerman 2010), scholars have yet to consider the more nuanced processes driving these differences.

Drawing from the work of Wikstrom and Sampson (2003), neighborhoods can be thought of as both (1) developmental contexts that influence the formation impulsive and violent tendencies and (2) social contexts which provide the opportunity for impulsivity to manifest in violent behavior. In this sense, the stronger association between impulsivity and violence in disadvantaged neighborhoods can be attributed to either the higher levels of violence and impulsivity among youth who reside in economically disadvantaged areas (a compositional effect) or the stronger effect on impulsivity on violence in these areas (a contextual effect). In an effort to disentangle these complimentary processes, this article applied a neighborhood-based, group decomposition. The results of the regression models indicate that impulsivity was positively associated with self-reported violence and that this association was strongest among youth residing in the most socioeconomically disadvantaged neighborhoods. The results of the decomposition reveal that much of the stronger effect of impulsivity on violence in disadvantaged neighborhoods could be attributed to contextual processes. In other words, there is something unique about socioeconomically disadvantaged neighborhoods that increased the effect of impulsivity on violence. However, a nontrivial portion of the interaction could be attributed to higher levels of impulsivity and self-reported violence among youth residing in disadvantaged areas, suggesting the moderating relationship uncovered in prior research reflects more than an abundance of opportunities for impulsive youth to offend in socioeconomically disadvantaged areas. Instead, there is strong evidence that both compositional and contextual processes are at play. In this sense, the present article provides a more nuanced framework for understanding the complex relationships between individual risk-factors and neighborhood features on adolescent development and behavior.

The decomposition techniques presented here provide a relatively intuitive means to bolster claims about the developmental and contextual underpinnings often assumed in person-context models of behavior. As such, we encourage researchers to consider such techniques in their own work. Insofar as there is apriori reason to assume compositional differences between groups, it would be useful to demonstrate the extent to which these differences drive interaction effects. We caution researchers from concluding they have uncovered evidence of contextual moderation when compositional differences account for the majority of the observed difference across groups. However, we also encourage researchers to present results in which compositional factors are primarily responsible for these differences, as the mechanisms driving compositional effects are meaningful in and of themselves.

The techniques presented here are not limited to studies examining the moderating role of neighborhood context on the association between impulsivity and violence; rather, they are a useful resource for researchers interested in theoretical models of behavior combining individual and contextual factors more generally. These techniques could be used to examine, for instance, the contribution of student composition to differences in victimization experiences, or to partition gene X environment interactions into compositional and environmental components. These techniques can be applied to most analyses examining interaction effects in which developmental processes generate meaningful compositional differences across groups.

It is important to note that such decomposition procedures reflect an exercise in variance partitioning. While these analyses provide some insight into the structure of the interaction effects, they do little to expound the causal processes underlying the stronger effect of impulsivity on violence in disadvantaged neighborhoods. Thus, these procedures allow us to conclude with relative certainty that the tract-level interactions are not statistical artifacts; however, the mechanisms underlying these effects remain to be determined. Likewise, we do not present this approach as an alternative to correcting regression models for endogeneity. Researchers who want to remove the confounding effects of composition remain well-served to employ standard counterfactual models, such as fixed-effects regressions, propensity score models, or instrumental variable approaches.

We would be remiss not to reiterate several key limitations of the findings reported here. The most glaring limitation is our operationalization and measurement of impulsivity as a single item, rather than a more comprehensive inventory that more fully captures into the multifaceted nature of the construct. As noted, the Add Health study was not designed to measure complex psychological traits. As a result, we were limited in the variables at our disposal. The assorted issues with measuring multifactorial constructs with national survey data are well-documented in the empirical literature (e.g., Wolfe and Hoffmann 2016). The use of imprecise measures from questionnaires that were not designed capture these traits pose a limitation to any study drawing from nationally representative survey data. As such, we would strongly encourage future researchers to replicate the results here with a more comprehensive psychometric measure of impulsivity that more closely captures each of its constituent dimensions (e.g., risk seeking, urgency, lack of perseverance).

The limitations of decomposition techniques have been well documented in the econometric literature (e.g., Jones 1983; Jones and Kelley 1984; Lin 2007), but warrant some discussion here. First, the results of these procedures are contingent on the category chosen as the referent. In the application presented here, disadvantaged neighborhoods. The choice of the reference group will alter the decomposition procedure, as the choice of the base category will affect the estimation of the coefficients in the regression equation (and, as a result, the relative contribution of each component to the overall difference). Second, in the standard 2-component decomposition, the interpretation of the unexplained portion (e.g., the difference in slopes) is sensitive to scaling decisions and this component only has a meaningful interpretation for variables which have a natural zero point (Jones and Kelley 1984). This issue, however, is resolved in the three—and four—component decompositions. Third, the procedure presented here assumed a binary moderator, in this case comparing disadvantaged and non-disadvantaged neighborhoods. Of course, collapsing continuous variables into dichotomies truncates meaningful variation in neighborhood disadvantage. To address this issue, researchers could employ the same framework and decompose the differences at various points of the neighborhood disadvantage index (e.g., one and two standard deviations above/below the mean). Fourth, the decomposition utilizes point estimates, thus ignoring the standard error of the coefficients. Although a bit beyond the purview of the present analysis, a handful of scholars have proposed ways to incorporate standard errors into the traditional decomposition framework (e.g., Lin 2007). Finally, and perhaps most importantly, the decomposition does not provide leverage to determine what is driving the difference in slopes, only the extent to which mean levels of X and Y contribute to the observed interaction.

## Conclusion

The results presented in this study underscore the complex pathways through which individual and contextual factors operate to influence adolescent behavior. This study demonstrated the extent to which variation in the association between impulsivity and delinquency across neighborhoods can be attributed to (1) differences in mean-levels of impulsivity and violence and (2) differences in coefficients across neighborhoods. The decomposition method showed that the differential effect of impulsivity on violence can be attributed to both developmental processes that lead to the greater concentration of violent and impulsive adolescents in economically deprived neighborhoods as well as the greater likelihood of impulsive adolescents engaging in violence when they reside in economically disadvantaged communities. We encourage future researchers to consider the nuanced role of developmental and contextual processes that link individual risk-factors to broader contextual influences. To this end, the neighborhood-based, group decomposition presented here is a useful heuristic tool for researchers interested in the direct and moderating effects of contextual influences on adolescent behavior. While the approach is commonplace in other social science disciplines, the decomposition framework is rarely utilized in person-context research. Unlike many of the methods du jour, this technique is relatively intuitive, computationally straightforward, and does not necessitate complex modeling strategies. In regards to the person-context literature in particular, we encourage researchers to simultaneously consider developmental and contextual influences in theoretical models linking individual behavior to broader social ecologies, and caution readers against placing too much stock in one mechanism without considering the contribution of the other. The decomposition framework provides a useful means to this goal.

## Appendix

Tables 6 and 7

**Table 6:** Summary statistics of impulsivity and violence across neighborhood type with 90/10 dichotomy

	Disadvantaged tracts	Non-disadvantaged tracts	Difference
Mean impulsivity	1.53	1.13	0.40
Impulsivity slope	3.11	3.00	0.11
a	0.20	0.09	0.12
Y-hat	0.90	0.87	0.04

**Table 7:** Decomposition of difference in impulsivity—violence association across neighborhood type

Component		Raw	Percent
Total difference	(ŷ_H_–ŷ_L_)	0.40	100.00%
Portion explained by differences in mean violence	(a_H_–a_L_)	0.04	9.15%
Portion explained by differences in mean impulsivity	b_L_(x̅_H_–x̅_L_)	0.01	2.39%
Portion explained by differences in impulsivity slope	x̅_L_(b_H_–b_L_)	0.35	85.33%
Residual difference	(b_H_–b_L_)(x̅_H_–x̅_L_)	0.01	3.12%

## Footnotes

1 Unfortunately the Add Health study does not include a comprehensive psychometric inventory and therefore we are limited in our ability to use a comprehensive measure of impulsivity. We should note that this item closely parallels a lack of premeditation, or an inability to think through the consequences of ones’ action, which has been established as a robust correlate of criminal offending (e.g., Gottfredson and Hirschi 1990). As a sensitivity analysis, we also re-estimated our models with alterative measures of impulsivity used in prior Add Health research (Vazsonyi et al. 2006; Perrone et al. 2004; McGloin and O’Neill Shermer 2009). The results of these supplemental models are available upon request from the corresponding author.

2 Some studies employ multilevel or hierarchical linear models (HLMs) to partition the variance in the dependent variable between individuals and neighborhoods. Unlike the methods presented here, HLM models require (1) nested data—that is, respondents clearly situated within broader neighborhood units, (2) a large number of neighborhoods, and a (3) large number of respondents in each neighborhood. Few extant data sources meet this requirement. Moreover, the models require that variance can be specified on both L1 and L2, which is not possible with count or categorical models—those most commonly employed in criminological research (Sweeten 2012). Thus, the strategy presented here is an alternative to the HLM framework when these computational requirements cannot be met.

3 Notably, similar strategies have also been applied in the criminological literature to examine differences in offending between males and females (e.g., Botchkovar and Broidy 2013; Botchkovar et al. 2015), and racial/ethnic differences in arrest (Kirk 2008) and incarceration (Vogel and Porter 2016).

4 Z = (b_H_−b_L_)/√(se_H_^2^ + se_L_^2^)

5 This example applies to a regression model in which neighborhood disadvantage is collapsed into a dichotomy. The same general framework can be applied to ordinal or continuous measures of neighborhood disadvantage. This extension would require expanding the number of regression equations to equal the number of categories, K, of the neighborhood measure. The decomposition could then be replicated by comparing Y and X for each of the possible pairwise comparisons, selecting the neighborhood with the highest level of disadvantage as the referent.

6 The decomposition presented here assumes the parameter estimates are generated from an OLS regression. This approach has also been expanded to apply to binary outcomes (Fairlie 1999, 2005), quantile regression, and counterfactual models (Machado and Mata 2005). Bauer and Sinning (2008) provide a generalized decomposition for non-linear models with discrete and limited dependent variables. The NLDECOMPOSE routine in Stata can handle these more complex modeling strategies (Sinning et al. 2008; Jann 2008).
